# Does hormonal therapy improve sperm retrieval rates in men with non-obstructive azoospermia: a systematic review and meta-analysis

**DOI:** 10.1093/humupd/dmac016

**Published:** 2022-05-08

**Authors:** Tharu Tharakan, Giovanni Corona, Daniel Foran, Andrea Salonia, Nikolaos Sofikitis, Aleksander Giwercman, Csilla Krausz, Tet Yap, Channa N Jayasena, Suks Minhas

**Affiliations:** Department of Urology, Imperial Healthcare NHS Trust, Charing Cross Hospital, London, UK; Department of Metabolism, Digestion and Reproduction, Imperial College London, London, UK; Endocrinology Unit, Medical Department, Azienda Usl Bologna Maggiore-Bellaria Hospital, Bologna, Italy; Department of Metabolism, Digestion and Reproduction, Imperial College London, London, UK; Department of Experimental Oncology/Unit of Urology, URI, IRCCS Ospedale San Raffaele, Milan, Italy; Department of Urology, University Vita-Salute San Raffaele, Milan, Italy; Department of Urology, Ioannina University School of Medicine, Ioannina, Greece; Department of Translational Medicine, Lund University, Lund, Sweden; Department of Experimental and Clinical Biomedical Sciences, University Hospital of Careggi (AOUC), University of Florence, Florence, Italy; Department of Urology, Guy’s and St Thomas’ Hospital, London, UK; Department of Metabolism, Digestion and Reproduction, Imperial College London, London, UK; Department of Urology, Imperial Healthcare NHS Trust, Charing Cross Hospital, London, UK

**Keywords:** non-obstructive azoospermia, testicular extraction sperm surgery, hypergonadotropic hypogonadism, selective oestrogen receptor modulators, aromatase inhibitors, gonadotrophins

## Abstract

**BACKGROUND:**

The beneficial effects of hormonal therapy in stimulating spermatogenesis in patients with non-obstructive azoospermia (NOA) and either normal gonadotrophins or hypergonadotropic hypogonadism prior to surgical sperm retrieval (SSR) is controversial. Although the European Association of Urology guidelines state that hormone stimulation is not recommended in routine clinical practice, a significant number of patients undergo empiric therapy prior to SSR. The success rate for SSR from microdissection testicular sperm extraction is only 40–60%, thus hormonal therapy could prove to be an effective adjunctive therapy to increase SSR rates.

**OBJECTIVE AND RATIONALE:**

The primary aim of this systematic review and meta-analysis was to compare the SSR rates in men with NOA (excluding those with hypogonadotropic hypogonadism) receiving hormone therapy compared to placebo or no treatment. The secondary objective was to compare the effects of hormonal therapy in normogonadotropic and hypergonadotropic NOA men.

**SEARCH METHODS:**

A literature search was performed using the Medline, Embase, Web of Science and Clinicaltrials.gov databases from 01 January 1946 to 17 September 2020. We included all studies where hormone status was confirmed. We excluded non-English language and animal studies. Heterogeneity was calculated using *I*^2^ statistics and risk of bias was assessed using Cochrane tools. We performed a meta-analysis on all the eligible controlled trials to determine whether hormone stimulation (irrespective of class) improved SSR rates and also whether this was affected by baseline hormone status (hypergonadotropic versus normogonadotropic NOA men). Sensitivity analyses were performed when indicated.

**OUTCOMES:**

A total of 3846 studies were screened and 22 studies were included with 1706 participants. A higher SSR rate in subjects pre-treated with hormonal therapy was observed (odds ratio (OR) 1.96, 95% CI: 1.08–3.56, *P* = 0.03) and this trend persisted when excluding a study containing only men with Klinefelter syndrome (OR 1.90, 95% CI: 1.03–3.51, *P* = 0.04). However, the subgroup analysis of baseline hormone status demonstrated a significant improvement only in normogonadotropic men (OR 2.13, 95% CI: 1.10–4.14, *P* = 0.02) and not in hypergonadotropic patients (OR 1.73, 95% CI: 0.44–6.77, *P* = 0.43). The literature was at moderate or severe risk of bias.

**WIDER IMPLICATIONS:**

This meta-analysis demonstrates that hormone therapy is not associated with improved SSR rates in hypergonadotropic hypogonadism. While hormone therapy improved SSR rates in eugonadal men with NOA, the quality of evidence was low with a moderate to high risk of bias. Therefore, hormone therapy should not be routinely used in men with NOA prior to SSR and large scale, prospective randomized controlled trials are needed to validate the meta-analysis findings.

## Introduction

Non-obstructive azoospermia (NOA) is the absence of sperm in the ejaculate secondary to impaired spermatogenesis (Schlegel, 2004) and represents the most severe form of male infertility. NOA is estimated to affect 1% of the male population and 10–20% of patients presenting with infertility (Jarow *et al.*, 1989). Biochemical hypogonadism is present in almost half of all patients with NOA (Bobjer *et al.*, 2012; Reifsnyder *et al.*, 2012).

The use of hormone therapy in men with NOA and hypergonadotropic hypogonadism (i.e. primary hypogonadism) or eugonadism is controversial (Kim and Schlegel, 2008; Reifsnyder *et al.*, 2012; Kumar, 2013; Shiraishi, 2015) with mixed outcomes reported in the literature although it is widely practiced.

Intratesticular testosterone (ITT) is required for spermiogenesis and serum testosterone has been shown to be an inaccurate surrogate for ITT level with differences ranging from 40- to 181-fold (Jarow *et al.*, 2001; McLachlan, 2002; Coviello *et al.*, 2004; Roth *et al.*, 2010).

In hypergonadotropic hypogonadism, both human and animal data suggest a pathological desensitization of the FSH receptor (FSHR) caused by high circulating levels of gonadotrophins (Gnanaprakasam *et al.*, 1979; Namiki *et al.*, 1985, 1987; Themmen *et al.*, 1991; Foresta *et al.*, 2004). It has been postulated that hormone therapy may benefit patients with hypergonadotropic hypogonadism by using GnRH to suppress gonadotrophin levels and thereby overcoming Sertoli cell receptor desensitization caused by chronically raised FSH levels (Foresta *et al.*, 2004, 2009). Foresta *et al.* (2009) conducted a randomized controlled trial (RCT) in hypergonadotropic men in which treatment with GnRH to induce hypogonadotropism followed by recombinant LH and FSH improved semen parameters and pregnancy rates.

The existence of a testosterone independent pathway for spermatogenesis, through supraphysiological FSH stimulation, provides a rationale for hormone stimulation therapy in both eugonadal and hypergonadotropic hypogonadism patients (Huhtaniemi, 2018; Oduwole et al., 2018a,b). Oduwole *et al.* (2018b) observed that constitutively activating FSHR mutations in mice were able to maintain spermatogenesis even in the absence of androgen signalling including treatment with the anti-androgen Flutamide. Furthermore, a case report (Gromoll *et al.*, 1996) of a male with an FSHR-D567G mutation who exhibited normal spermatogenesis after hypophysectomy suggests that a strong constitutive FSH stimulation can compensate for a deficiency in LH and testosterone.

The current European Association of Urology (EAU) guidelines on Male Sexual and Reproductive Health do not advocate hormone stimulation therapy in idiopathic NOA (Salonia et al., 2021). However, a survey reported that 64.9% of urologists prescribe empiric hormone therapy to treat idiopathic male infertility, with clomiphene citrate the most commonly prescribed drug for both general and fertility-trained urologists (Ko *et al.*, 2012). This may be attributable to the fact that surgical sperm retrieval (SSR) rates in patients with NOA have remained static (40–60%) over the last 10 years (Shiraishi *et al.*, 2012; Corona *et al.*, 2019). Therefore, hormone therapy has been proposed as an adjunctive therapy to improve fertility outcomes (i.e. SSR rates and production of sperm into the ejaculate) in men with NOA.

This is the first systematic review and meta-analysis to investigate the effects of hormone therapy on SSR rate. The primary outcome of the meta-analysis was the SSR rate in men with NOA who were treated with hormone therapy. The secondary outcome was comparison of SSR rates according to baseline hormone status (hypergonadotropic versus normogonadotropic NOA men).

## Methods

This systematic review and meta-analysis was conducted according to the Preferred Reporting Items for Systematic reviews and Meta-analyses (PRISMA) guidelines and was registered in the international prospective register of systematic reviews (PROSPERO, ID CRD42019145226).

### Literature search

A literature search was performed using the Medline, Embase, Web of Science and Clinicaltrials.gov databases from 01 January 1946 to 17 September 2020. Search terms included: azoospermia, selective oestrogen receptor modulators, tamoxifen, clomiphene, gonadotropins, gonadotropin releasing hormone, aromatase inhibitors, anastrozole, letrozole, testolactone, chorionic gonadotropin, human chorionic gonadotropin, menotropins, human menopausal gonadotropin, sperm retrieval, testicular sperm extraction, microdissection testicular sperm extraction, testicular sperm aspiration and the corresponding abbreviations.

### Inclusion and exclusion criteria

For the systematic review, we included prospective and retrospective case series, case-control studies and RCTs. Studies for possible inclusion needed to confirm subjects with NOA and the hormone status of the participants and the type(s) and duration of hormone treatment. Non-English language and animal studies were excluded. We included abstracts and full-text studies. There were no age restrictions, and we included all patients with NOA irrespective of genetics status. In the case of multiple publications with overlapping cohorts, we included only the most recent study unless specified otherwise. For the meta-analysis, we only included controlled studies. We included multiple cohort studies when one arm fulfilled the aforementioned criteria.

### Study selection

Screening of the study abstracts was performed by two independent reviewers (T.T. and D.F.). Any discrepancy was discussed, and consensus achieved by a third reviewer (C.N.J.). Full-text articles were retrieved and underwent further utility assessment by two independent reviewers (T.T. and D.F.) with any differences being adjudicated by a third reviewer (S.M.). In cases where outcome measures were absent from the full-text article, the authors of the study were contacted to provide the raw data.

### Outcomes and quality assessment of included studies

There is no reference gonadotrophin or testosterone level to achieve optimal spermatogenesis in men with either eugonadism or with hypergonadotropic hypogonadism. We therefore compared the differences in serum testosterone, FSH and LH among each type of hormone treatment where applicable. For the purpose of the systematic review, we accepted mean or median cohort testosterone values as a representation of overall cohort hormone status. A successful sperm retrieval was defined as the presence of a single spermatozoon or more. Conventional testicular sperm extraction (TESE) was defined as single or multiple random biopsies of the testicular tissue while microdissection TESE was defined as TESE under magnification utilizing the technique previously described by Schlegel (1999).

Where indicated, hormone status was defined according to the reference ranges utilized in each individual study or authors descriptions of hormone status (e.g. normal hormone profiles). In cases of ambiguity, the authors were contacted for clarification and in the absence of a response, an FSH level of ≥12 mUI/ml and an LH ≥10 mUI/ml was used to define hypergonadotropic hypogonadism as these were the most common (mode) upper limit thresholds utilized in all the included studies. Similarly, hypogonadism was defined as a serum testosterone level <8.8 nmol/l as this was the average (mean) lowest reference threshold for hypogonadism in the included studies. If a single gonadotrophin was raised (FSH or LH) than this was categorized as hypergonadotropic. In addition to this, men with a raised FSH or LH and a normal testosterone were classified as compensated hypergonadotropic hypogonadism.

Full-text articles were studied, and the outcome measures recorded included baseline hormone parameters, type and duration of hormone agent, type of surgery, SSR rates, sperm production in the ejaculate and adverse events.

The risk of bias was evaluated using the ROBINS-1 tool (Sterne *et al.*, 2016) for non-RCTs (Aydos *et al.*, 2003; Hussein *et al.*, 2013; Gul, 2016; Cocci *et al.*, 2018; Hu *et al.*, 2018) included in the meta-analysis. Two reviewers (T.T. and D.F.) performed independent assessments of risk of bias with discrepancies being resolved by a third reviewer (S.M.).

### Meta-analysis and statistical analysis

Only controlled studies were included for the meta-analysis. We pooled data and performed a meta-analysis of all controlled trials to determine whether hormone stimulation (irrespective of class) improved SSR rates in hypergonadotropic men with NOA and eugonadal men with NOA. We also studied whether hormone therapy improved the SSR rate overall (irrespective of hormone status). Sensitivity analyses were performed when indicated.

Heterogeneity in SSR was assessed using *I*^2^ statistics. Even when low heterogeneity was detected, a random-effect model was applied because the validity of tests of heterogeneity can be limited with a small number of component studies. We used funnel plots and the Begg adjusted rank correlation test to estimate possible publication or disclosure bias (Begg and Mazumdar, 1994); however, undetected bias may still be present, because these tests have low statistical power when the number of trials is small. Overall SSR is expressed as a mean percentage (95% CI). All data were calculated using the Comprehensive Meta-analysis Version 2, Biostat, and (Englewood, NJ, USA). a value of *P* < 0.05 was considered significant.

## Results

### Evidence synthesis

**Figure 1.**
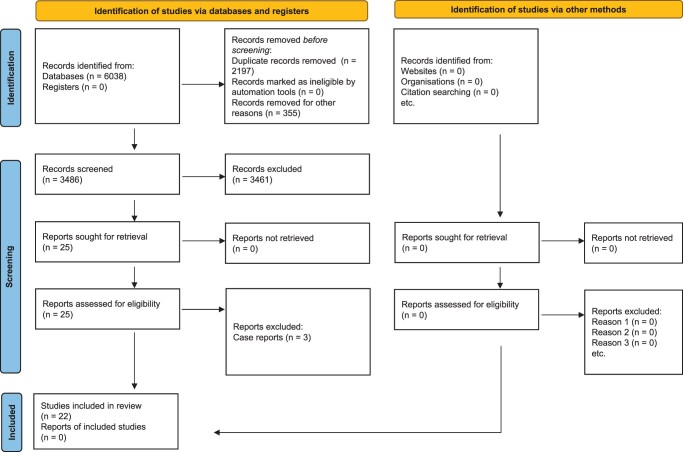
**PRISMA flow chart for the selection of studies on hormone therapy and sperm retrieval rates in men with non-obstructive azoospermia.** PRISMA, Preferred Reporting Items For Systematic Reviews and Meta-analysis.

Figure 1 shows the PRISMA flow-chart of the studies. We screened 3846 studies and included 22 studies of which 10 were case-control studies, 11 were case series and 1 was an RCT.

**Figure 2.**
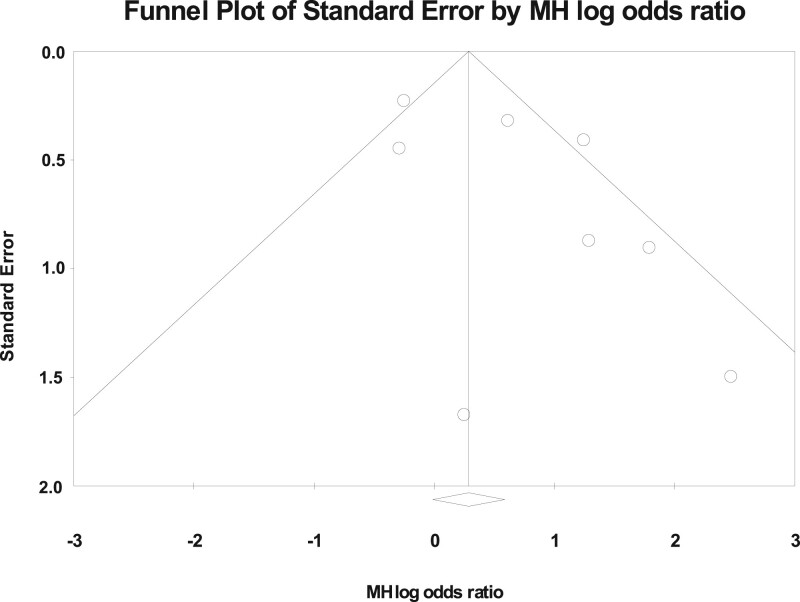
**A funnel plot of standard error of sperm retrieval rate by Mantel–Haenszel log odds ratio.** MH, Mantel–Haenszel.

For the purposes of the systematic review, we subdivided the cohorts of NOA into hypergonadotropic hypogonadism (Table I) and eugonadism (Table II). Any study which included a mixture of eugonadal and hypergonadotropic hypogonadism patients were analysed separately (Table III).

**Table I:** Studies assessed in the systematic review that evaluated the use of hormone stimulation therapy in men with non-obstructive azoospermia and hypergonadotropic hypogonadism.

Study (year)	Design	Population	Genetics	Mean age (SD) (*range) *in years* **=median	Intervention regime	Type of surgery	Hormone changes after hormone therapy	Rates of sperm returning to the ejaculate/surgical sperm retrieval (patients with NOA only)	**Pregnancy Live birth rates**	Adverse events	Strengths	Limitations
Shiraishi *et al.* (2012)	Case control	cHH NOA (n = 48) Intervention (n = 28) Control (n = 20)	Chromosomal abnormalities excluded	34 (5.7)	5000 IU hCG 3 times a week for 4–5 months (n = 13) ***or*** 5000 IU hCG 3 times a week for 5 months ***and*** 150 IU FSH 3 times a week for 2 months (n = 15) Control group: no treatment	Secondary mTESE	hCG only cohort: Increased tT from baseline (*P* < *0.01*) Decreased LH from baseline (*P* < *0.05*) FSH unchanged	SSR via mTESE: Intervention group: 6/28 (21.4%) Control group: 0/20 (0%) (*P* < 0.05) Increased SSR associated with hypospermatogenesis (*P* < 0.05)	NR	Acne: 3/28 (10.7%) Gynecomastia 2/28 (7.1%)	• Control included	Retrospective Risk of selection bias All patients had previously failed TESE Variable additional FSH treatment given to some patients based on hormone measurement Inadequately powered for all aspects of the analysis Pregnancy/live birth rates NR
							hCG and FSH cohort: Increased tT from baseline (*P* < *0.0001*) - Decreased LH and FSH from baseline (both *P* < *0.0001*)					

Shiraishi *et al.* (2016)	Case series	cHH NOA (n = 21)	Chromosomal abnormalities excluded	32.2 (3.1) (*29–36)	5000 IU hCG 3 times a week for 4 months ***and*** 150 IU FSH 3 times a week for 3 months Total duration: 4 months	Secondary mTESE	Increased tT and E2 from baseline (both *P* *< 0.01*) Decreased FSH and LH from baseline (both *P* *< 0.01*)	SSR via mTESE: 2/21 (9.5%) Increased SSR associated with hypospermatogenesis and late maturation arrest (*P* < 0.01)	PR: 1/21 (4.8%) LBR: 1/21 (4.8%)	Acne: 3/21 (14.3%)	• Pregnancy/live birth rates measured	Retrospective No control

Hu *et al.* (2018)	Case control	cHH NOA (n = 35) Intervention (n = 25) Control (n = 10)	Chromosomal abnormalities excluded	Intervention group: 25.8 (3.4) Control group: 26.6 (3.3)	3.6 mg Goserelin once every 4 weeks for 6 months ***and*** 2000 IU hCG once a week for 5 months ***and*** 150 IU hMG twice a week for 4 months Control group: no treatment	Secondary mTESE	Intervention group: Increased tT from baseline (*P* *< 0.05*) Decreased FSH and LH from baseline (both *P* *< 0.001*)	Rate of sperm in the ejaculate: Intervention group: 1/25 (4%) Control group: 0/10 (0%) Mean sperm concentration: 1.42 ×10^6^/ml Mean total sperm count: 3.98 ×10^6^ SSR via mTESE: Intervention group: 1/25 (4%) Control group: 0/25 (0%)	NR	Symptoms of androgen deprivation (e.g erectile dysfunction) on Goserelin: 10/25 (40%) Resolved with hCG Did not tolerate treatment: 10/25 (40%)	• Control included	Retrospective Risk of selection bias Pregnancy/live birth rates NR Controls were men who didn’t tolerate therapy; both groups were treated Subjects stratified into subgroups for analysis Unclear whether statistically significant difference in SSR outcomes

Pavlovich *et al.* (2001)	Case series	HH NOA (n = 43) ***and*** Oligospermia (n = 20)	Chromosomal abnormalities included	37 (*31–43)	Testolactone 50 mg twice daily for mean duration 5 months If oestradiol still high after 1 month then testolactone 100 mg twice daily Mean treatment duration: 5 months	Not applicable	Increased mean tT (*P* *< 0.01*) and T:E (*P* *< 0.01*) from baseline Decreased mean E2 (*P* *< 0.01*) from baseline	Rate of sperm in the ejaculate: 0/12	NR	Asymptomatic deranged Liver function tests 8/43 (18.6%) - Resolved on cessation of therapy		Retrospective Pregnancy/live birth rates NR No control No distinction between oligospermia and NOA Semen analysis for only 12 men No SSR attempt Variable treatment duration Chromosomal abnormalities included

Saylam *et al.* (2011)	Case series	HH NOA (n = 17) ***and*** Oligospermia (n = 10) (all T:E < 10)	NR	34.92 (6.66) (*26–49)	Letrozole 2.5 mg once daily for ≥6 months Mean treatment duration: 6.59 ± 0.88 months	Not applicable	Increased tT and T:E from baseline (*P* *= 0.001*) Decreased E2 from baseline (*P* *= 0.001*) LH and FSH no change	Rate of sperm in the ejaculate: 4/17 (23.5%)	NR	Mild headaches: 2/27 (7.4%)		Retrospective Pregnancy/live birth rates NR No control No distinction between oligospermia and NOA No SSR attempt Variable treatment duration

Cavallini *et al.* (2013)	RCT	HH NOA (n = 11) ***and*** Cryptospermia (n = 35) Intervention (n = 22) *HH NOA (n = 6)* *Cryptospermia (n = 16)* Control (n = 24) *HH NOA (n = 5)* *Cryptospermia (n = 19)*	Chromosomal abnormalities excluded	Intervention group: 44 (*37–52) Control group: 45 (*38–53)	Letrozole 2.5 mg once daily for 6 months Control group: placebo	Not applicable	Intervention group: Increased tT, FSH, and LH at 3 and 6 months (all *P* *< 0.01*) Control group: no change	Rate of sperm in the ejaculate: Intervention group: 6/6 (100%) Control group: 0/5 (0%)	PR: 0/46 (0%)	Loss of libido, loss of hair, + cutaneous rash: 4/22 (18.2%) Dropped out of study	Prospective Patients randomized Double blinded Control included Pregnancy/live birth rates measured Modified intention to treat analysis	No distinction between oligospermia and NOA Attrition due to side effects Small cohort No SSR attempt
Shoshany *et al.* (2017)	Case series	HH NOA (n = 28) ***and*** Men with normal and abnormal semen parameters (n = 58)	Chromosomal abnormalities excluded	**37 (*32–41)	Anastrazole 1 mg once daily for 4 months	Primary mTESE	Increased LH, FSH, tT, and T:E at 3 weeks (all *P* < *0.0001*) Decreased E2 at 3 weeks (*P* < *0.0001*)	Rate of sperm in the ejaculate: 0/28 SSR via mTESE (n = 11) 8/11 (72.7%) 17/28 did not undergo surgery	NR	Joint pain, lower limb swelling, low libido, ocular pruritus/pain, depression, mastalgia, + dry mouth: 8/86 (9.3%) Treatment stopped in affected patients		Retrospective Pregnancy/live birth rates NR No control No distinction between oligospermia and NOA SSR only done on 39% of patients Attrition due to side effects

Reifsnyder *et al.* (2012)	Case control	HH NOA (n = 348) Intervention (n = 307) Control (n = 41)	Exclusion of azoospermia factor gene a, b and c Y microdeletion Included some chromosomal abnormalities, i.e. Klinefelter syndrome	35	Regimes unspecified anastrozole (n = 180) Anastrozole + hCG (n = 29) CC (n = 66) Testolactone (n = 14) Testolactone + hCG (n = 12) hCG (n = 9) Other combinations/unknown (n = 38) Minimum treatment duration: 2–3 months Control group: mTESE only	Primary mTESE	Decreased post-treatment FSH in intervention group compared to control (*P* *= 0.02*)	SSR via mTESE Intervention group: 157/307 (51.1%) Control group: 25/41 (61.0%) (*P* *= 0.31*) No association between SSR and response to therapy in intervention group (resultant tT >250 ng/dl) (*P* = 0.97)	No significant difference in, PR and LBR	NR	Control included Large cohort size Pregnancy/live birth rates measured	Retrospective Risk of selection bias Incl. cohort w. unknown treatment regimens Combination of different drug classes within groups Incomplete chromosomal abnormality exclusion Variable treatment duration; not defined Some of the cohort had pre-treatment Analysis did not control for different drug classes

Majzoub *et al.* (2016)	Case control	HH NOA (n = 20) Intervention (n = 16) *-Group A1 (n = 10)* *-Group A2 (n = 6)* Control (n = 4)	All subjects: non-mosaic Klinefelter syndrome Exclusion of azoospermia factor gene a, b and c Y microdeletion	32.9	Intervention group: Group A1: Anastrozole 1 mg once daily, 6 months Group A2: CC 25 mg once daily and hCG 5000 IU once weekly (no treatment duration specified) Control group: no treatment	Primary mTESE	Statistically significant increase in testosterone in intervention group compared to controls (*P* *= 0.01*), but no difference in FSH and LH	SSR via mTESE Intervention group: 6/16 (37.5%) Control group: 0/4 (0%)	PR: 3/16 (18.8%) LBR: 3/16 (18.8%)	NR	Control included Pregnancy/live birth rates measured Exclusion of AZF Y mutations Histology controlled	Retrospective Risk of selection bias Combination of different drug classes within groups Patients are all Klinefelters Treatment duration not defined Unclear whether differences in SSR was statistically significant

Amer *et al.* (2020)	Case control	HH NOA (n = 40) Intervention (n = 20) Control (n = 20)	NR	Intervention group: 36.2 (4.3) Control group: 35.9 (5.4)	250 mg testosterone enanthate once a week for 1 month Then 5000 IU hCG once a week, 150 IU puFSH thrice a week, and 250 mg testosterone enanthate once a week for 3 months	Secondary mTESE	NR	SSR via mTESE: Intervention group: 2/20 (10%) Control group: 0/20 (0%) (*P* = 0.072)	NR	NR	Prospective Control included	Retrospective -Risk of selection bias Pregnancy/live birth rates NR Hormone changes NR Testosterone use

Sujenthiran *et al.* (2019)	Case series	HH NOA (n = 23) Intervention (n = 15) Control (n = 8)	All subjects: Klinefelter syndrome	**33 (IQR 30–34)	Intervention group: CC or hCG and FSH. Treatment duration: 6 months Control group: no treatment	NR	NR	SSR via mTESE: Intervention group: 6/15 (40%) Control group: 1/8 (13%)	Intervention group: PR: 4/15 (26.7%) LBR: 3/15 (20%)	NR	• Pregnancy/live birth rates measured	Retrospective Hormone changes NR No control Patients are all Klinefelters Treatment duration not defined

CC, clomiphene citrate; cHH, compensated hypergonadotropic hypogonadism; E2, serum oestrogen; HH, hypergonadotropic hypogonadism; IQR, interquartile range; LBR, live birth rate; mTESE, microtesticular sperm extraction; NOA, non-obstructive azoospermia; NR, not reported; PR, pregnancy rate; puFSH, purified urinary FSH; RCT, randomized control trial; SSR, successful surgical sperm retrieval; T:E, testosterone oestrogen ratio; tT, serum total testosterone.

**Table II:** Studies assessed in the systematic review that evaluated the use of hormone stimulation therapy in eugonadal men and non-obstructive azoospermia.

**Study (year)**	Design	Population	Genetics	Mean age (SD) (*range) *in years* **=median	Intervention regime	Type of surgery	Hormone changes after hormone therapy	Rates of sperm returning to the ejaculate/surgical sperm retrieval (patients with NOA only)	Pregnancy Live birth rate	Adverse events	Strengths	Limitations
Aydos *et al.* (2003)	Case control	NG NOA (n = 174) Intervention (n = 63) Control (n = 45)	Chromosomal abnormalities included	29 (*21–39)	Intervention: 75 IU FSH I.M. 3 times a week for 3 months Control group: no treatment	Primary cTESE	FSH increase in intervention group vs controls (*P* < *0.001*)	SSR via cTESE: Intervention group: 40/63 (63.5%) Control group: 15/45 (33.3%) No significant difference. Increased SSR was associated with cohorts with focal spermatogenesis and hypospermatogenesis (*P* < 0.05)	NR	No adverse effects observed	Control included Controlled for histology in analysis Large cohort size	Retrospective Risk of selection bias Pregnancy/ live birth rates NR cTESE used Chromosomal abnormalities included Data table printing error

Selman *et al.* (2006)	Case series	NG NOA (n = 49)	Chromosomal abnormalities excluded	(*32–41)	75 IU rFSH alternate days for 2 months 150 IU rFSH alternate days for 4 months From 4th month, hCG 2000 IU twice weekly for 2 months	Secondary cTESE	NR	Rate of sperm in the ejaculate: 0/49 (0%) SSR via cTESE: 11/49 (22.4%)	PR: 3/49 (6.1%) LBR: 3/49 (6.1%)	NR	• Pregnancy/live birth rates measured	Retrospective Hormone changes NR No control cTESE used

Efesoy *et al*.(2009)	Case series	NG NOA (n = 11)	NR	31.1 (4.52)	100–150 IU FSH 2–3 times a week Mean treatment duration (7.45 ± 4.5 months)	Primary mTESE	Increase in FSH (*P* = *0.004*)	Rate of sperm in the ejaculate: 2/11 (18.1%) (*P* = 0.323) SSR via mTESE: 2/11 (18.1%)	NR	No adverse events observed	• Prospective	No control Small cohort Variable treatment duration

Gul (2016)	Case control	NG NOA (n = 83) Intervention (n = 34) Control (n = 49)	Chromosomal abnormalities excluded	34 (5.7)	hCG 2500 IU twice a week for 10–14 weeks Control group: no treatment	Primary cTESE (and if this failed then mTESE)	NR	SSR via cTESE and mTESE: Intervention group: 17/34 (50%) Control group: 28/49 (57.1%) (*P* = 0.338)	No significant difference in FR, PR and LBR	No adverse events observed	Control included Pregnancy/live birth rates measured	Retrospective Risk of selection bias Hormone changes NR Patients have all failed previous TESE Variable treatment duration Variable TESE technique

Cocci *et al.* (2018)	Case control	NG NOA (n = 50) Intervention (n = 25) Control (n = 25)	NR	35.5 (4.3)	150 IU FSH, S.C. 3 times a week for 3 months Control group (retrospective cohort): no treatment	Primary cTESE	NR	Rate of sperm in the ejaculate: Intervention group: 5/25 (20%) Control group: 0/25 (0%) (*P* < 0.05)	Increased FR and PR in treated group vs controls (*P* < 0.05)	NR	Control included Pregnancy/live birth rates measured Controlled for testis volume	Retrospective Risk of selection bias Hormone changes NR cTESE used
								SSR via cTESE: Intervention group: 6/25 (24%) Control group: 2/25 (8%) (*P* < 0.05)				

Cavallini *et al.* (2011)	Case series	NG NOA (N = 4)	Chromosomal abnormalities excluded	37.3 (*29–44)	Letrozole 2.5 mg, orally, once daily for 6 months	Not applicable	Increases in tT, FSH and, LH (*P* < *0.05 for all*). Oestrogen decreased (*P* < *0.01*)	Rate of sperm in the ejaculate: 4/4 (100%)	NR	Loss of libido, Cutaneous rash, and anxiety		Retrospective Pregnancy/live birth rates NR No control Small cohort No SSR attempt

Hussein *et al.* (2013)	Case control	NGH NOA (n = 612) Intervention groups: (n = 496) *#1 (n = 372)* *#2 (n = 62)* *#3 (n = 46)* *#4 (n = 16) * Control (n = 116)	NR	26.7 (4.9)	Intervention groups: Different therapies based on initial response to CC. #1: CC (6.4 ± 2 months) #2: CC and hCG (4.1 ± 2.4 months) #3: hMG + hCG (4.2 ± 1.1 months) #4 hMG + hCG (4.2 ± 1.1 months) Control group: no treatment	Primary mTESE	All groups reached target tT level (600–800 ng/dl) FSH increased in all groups	Rate of sperm in the ejaculate: Intervention group 1: 41/372 (11.0%) (*P* < 0.001) Intervention group 2: 7/62 (11.3%) (*P* < 0.001) Intervention group 3: 4/46 (8.7%) Intervention group 4: 2/16 (12.5%) (*P* < 0.05) Control group: 0/116 (0%)	NR	Paradoxical decrease in serum tT level on CC: 16/496 (3.2%)	Control included Large cohort size	Retrospective Risk of selection bias Pregnancy/live birth rates NR All patients received CC pre-treatment prior to switch Combination of different drug classes within groups Variable treatment dose and duration SSR not performed in all patients
								SSR via mTESE: Intervention group 1: 191/331 (57.7%) (*P* < 0.001) Intervention group 2: 31/55 (56.3%) (*P* < 0.001) Intervention group 3: 22/42 (52.4%) Intervention group 4: 8/14 (57.1%) (*P* < 0.05) Control group: 39/116 (33.6%)				

Song and Qian (2012)	Case series	NG NOA (n = 4) ***and*** oligospermia (n = 8)	Chromosomal abnormalities excluded	(*25–39)	Testosterone undecanoate 40 mg twice daily and TC 10 mg twice daily for 4 months	Not applicable	Increase in FSH and LH (*P* < *0.01*)	Rate of sperm in the ejaculate: NOA patients: 4/4 (100%) Max duration for sperm to return to the ejaculate: 2 months	NR	NR		Retrospective Pregnancy/ live birth rates NR No control No distinction between oligospermia and NOA Use of testosterone Small cohort No SSR attempt

Sen *et al.* (2020)	Case control	NGH NOA (n = 24) Intervention: NGH (n = 12) Control: HH (n = 12)	NR	Intervention group: 36.58 (2.01) Control group: 41 (2.37)	250 mcg recombinant HCG once/week for 6 months. Control group: no treatment	Primary mTESE	Intervention group serum tT increased from 8.03 (±0.97) to 15.66 (±2.20)	Rate of sperm in the ejaculate: Intervention group: 3/12 (25%) Control group: 0/12 (0%)	NR	NR	• Control included	Retrospective Risk of selection bias Pregnancy/live birth rates NR
								SSR via mTESE: Intervention group: 6/12 (66.6%) Control group: 4/12 (33.3%) (*P* < 0.05)				

CC, clomiphene citrate; cTESE, conventional testicular sperm extraction; FR, fertilization rate; HH, hypergonadotropic hypogonadism; I.M., intramuscular injection; LBR, live birth rate; mTESE, microtesticular sperm extraction; NG, normogonadotropic eugonadism; NGH, normogonadotropic hypogonadism; NOA, non-obstructive azoospermia; NR, not reported; PR, pregnancy rate; rFSH, recombinant FSH; S.C., subcutaneous injection; SSR, successful surgical sperm retrieval; TC, tamoxifen citrate; tT, serum total testosterone.

**Table III:** Studies assessed in the systematic review that evaluated the use of hormone stimulation therapy in a mixed cohort of both eugonadal and hypergonadotrophic hypogonadism non-obstructive azoospermia men.

Study (year)	Design	Population	Genetics	Mean age (SD) (*range) *in years* **=median	Intervention regime	Type of surgery	Hormone changes	Rates of sperm returning to the ejaculate/surgical sperm retrieval (NOA patients only)	Pregnancy Live birth rate	Adverse events	Strengths	Limitations
Kumar *et al.* (1990)	Case series	NG and cHH NOA (n = 50) ***and***Oligospermia (n = 29)	Chromosomal abnormalities excluded	31 (4.7)	2000 units hCG, twice a week for 6 months ***Or***CC (50 mg once a day, 25 days per month for 6 months)	Not applicable	NR	Rate of sperm in the ejaculate: 0/50 (0%)	NA	NR		Retrospective Pregnancy/live birth rates NR Hormone changes NR No control No SSR attempt Mixed cohort

Kobori *et al.* (2015)	Case series	HH, cHH and NG NOA (n = 26)	Chromosomal abnormalities excluded	34.6 (*29–38)	75 IU FSH twice a week for the first 3 months, then 150 IU twice a week subsequently	Not applicable	NR	Rate of sperm in the ejaculate: 5/26 (19.2%) Mean concentration: <1 million/ml - Mean duration for sperm to return to the ejaculate: 4.4 months	PR: 2/26 (7.7%) LBR: 1/26 (3.9%)	NR	−Pregnancy/live birth rates measured	Retrospective Hormone changes NR Only reported data for the five patients who produced sperm in the ejaculate No control No SSR attempt Mixed cohort

CC, clomiphene citrate; cHH, compensated hypergonadotropic hypogonadism; HH, hypergonadotropic hypogonadism; LBR, live birth rate; NG, normogonadotropic eugonadism; NOA, non-obstructive azoospermia; NR, not reported; PR, pregnancy rate; SSR, successful surgical sperm retrieval.

### Men with non-obstructive azoospermia and hypergonadotropic hypogonadism

There have been 11 studies (Pavlovich *et al.*, 2001; Saylam *et al.*, 2011; Reifsnyder *et al.*, 2012; Shiraishi *et al.*, 2012, 2016; Cavallini *et al.*, 2013; Majzoub *et al.*, 2016; Shoshany *et al.*, 2017; Hu *et al.*, 2018; Sujenthiran *et al.*, 2019; Amer *et al.*, 2020) investigating the use of hormone therapy in men with NOA and primary hypogonadism. The literature predominantly consisted of case series (n = 5) (Pavlovich *et al.*, 2001; Saylam *et al.*, 2011; Shiraishi *et al.*, 2016; Shoshany *et al.*, 2017; Sujenthiran *et al.*, 2019) and case-control studies (n = 5) (Reifsnyder *et al.*, 2012; Shiraishi *et al.*, 2012; Majzoub *et al.*, 2016; Hu *et al.*, 2018; Amer *et al.*, 2020) with only one RCT (Cavallini *et al.*, 2013). There were four studies solely utilizing aromatase inhibitors (Pavlovich *et al.*, 2001; Saylam *et al.*, 2011; Cavallini *et al.*, 2013; Shoshany *et al.*, 2017), two studies investigating gonadotrophin therapy (Shiraishi *et al.*, 2012, 2016) and three studies investigating multiple hormone agents (aromatase inhibitors, gonadotrophins, selective oestrogen receptor modulators (SERM’s) and combinations e.g. aromatase inhibitors and hCG) (Reifsnyder *et al.*, 2012; Majzoub *et al.*, 2016; Sujenthiran *et al.*, 2019). Two studies investigated the use of gonadotrophins with an anti-gonadotrophin agent (either in the form of goserelin or exogenous testosterone) (Hu *et al.*, 2018; Amer *et al.*, 2020). The literature included three studies analysing patients undergoing primary TESE (Reifsnyder *et al.*, 2012; Majzoub *et al.*, 2016; Shoshany *et al.*, 2017), four studies investigated patients undergoing secondary TESE (Shiraishi *et al.*, 2012, 2016; Hu *et al.*, 2018; Amer *et al.*, 2020) and one study did not report the operation status (Sujenthiran *et al.*, 2019). There were three studies investigating only the effect of hormone therapy on NOA men producing sperm in their ejaculate (Pavlovich *et al.*, 2001; Saylam *et al.*, 2011; Cavallini *et al.*, 2013). There were five studies that excluded chromosomal abnormalities (Shiraishi *et al.*, 2012, 2016; Cavallini *et al.*, 2013; Shoshany *et al.*, 2017; Hu *et al.*, 2018), four studies included patients with these abnormalities (Pavlovich *et al.*, 2001; Reifsnyder *et al.*, 2012; Majzoub *et al.*, 2016; Sujenthiran *et al.*, 2019) and two studies did not report on genetic findings (Saylam *et al.*, 2011; Amer *et al.*, 2020). The treatment duration ranged from 2 to 6.5 months.

Of the case-control studies, the outcomes were variable; one study (Shiraishi *et al.*, 2012) investigating hCG and FSH showed a statistically significant improvement in SSR in those receiving hormone therapy compared to no treatment (21.4% versus 0%, respectively *P* < 0.05) while two studies (Reifsnyder *et al.*, 2012; Amer *et al.*, 2020) reported no significant differences in SSR between the treatment and control cohorts. Two studies (Majzoub *et al.*, 2016; Hu *et al.*, 2018) observed improved SSR outcomes with hormone stimulation compared to no treatment but no statistical significance analysis was performed. The single RCT observed that the use of aromatase inhibitors resulted in all NOA patients (n = 6) producing sperm in the ejaculate compared to zero in the control group who did not receive any hormone therapy (n = 6) but it is unclear whether this was statistically significant. The cause for these differences in outcomes is unclear but may be related to study heterogenicity with regards to the patient cohorts, operation status (primary versus secondary TESE) and treatment protocol.

Overall, the following adverse effects were reported with the use of hormone therapy: acne, gynaecomastia, deranged liver function tests, headache, loss of libido, hair loss, joint pain, cutaneous rash, lower limb swelling, ocular pruritus, depression, mastalgia and dry mouth. In three studies, the dropout rates owing to treatment side effects were 9.3% (Shoshany *et al.*, 2017), 18.2% (Cavallini *et al.*, 2013) and 40% (Hu *et al.*, 2018). The main limitation to the current literature is the lack of standardization in terms of treatment regimens and patient cohorts, few studies report pregnancy or live birth rates, and a large proportion of the data is retrospective, case series. Furthermore, there is no clear trend regarding whether hormone therapy improves SSR outcomes compared to no treatment or placebo.

### Men with non-obstructive azoospermia and eugonadism

There have been eight studies (Aydos *et al.*, 2003; Selman *et al.*, 2006; Efesoy *et al.*, 2009; Cavallini *et al.*, 2011; Song and Qian, 2012; Hussein *et al.*, 2013; Gul, 2016; Cocci *et al.*, 2018) investigating the use of hormone therapy in men with NOA and eugonadism. The literature consisted of case series (n = 4) (Selman *et al.*, 2006; Efesoy *et al.*, 2009; Cavallini *et al.*, 2011; Song and Qian, 2012) and case-control studies (n = 4) (Aydos *et al.*, 2003; Hussein *et al.*, 2013; Gul, 2016; Cocci *et al.*, 2018) with no RCTs. One study solely utilized aromatase inhibitors (Cavallini *et al.*, 2011), five studies investigated gonadotrophin therapy (Aydos *et al.*, 2003; Selman *et al.*, 2006; Efesoy *et al.*, 2009; Gul, 2016; Cocci *et al.*, 2018) and one study investigated multiple hormone agents (SERMs, gonadotrophins) (Hussein *et al.*, 2013). One study investigated the use of SERMs with exogenous testosterone (Song and Qian, 2012). The data included four studies analysing patients undergoing primary TESE (Aydos *et al.*, 2003; Efesoy *et al.*, 2009; Hussein *et al.*, 2013; Gul, 2016; Cocci *et al.*, 2018) and one study investigated patients undergoing secondary TESE (Selman *et al.*, 2006). There were two studies investigating only the effect of hormone therapy in men with NOA producing sperm in their ejaculate (Cavallini *et al.*, 2011; Song and Qian, 2012), and the treatment duration ranged from 3 to 7 months. There were four studies that excluded chromosomal abnormalities (Selman *et al.*, 2006; Cavallini *et al.*, 2011; Song and Qian, 2012; Gul, 2016), one study that included chromosomal abnormalities (Aydos *et al.*, 2003) and three studies that did not report on the genetic status of the participants (Efesoy *et al.*, 2009; Hussein *et al.*, 2013; Cocci *et al.*, 2018).

Of the case-control studies, the outcomes were inconsistent; two studies (employing gonadotrophins) did not show any statistically significant difference in SSR between those receiving hormone therapy and those proceeding straight to TESE (Aydos *et al.*, 2003; Gul, 2016). However, Cocci *et al.* (2018) observed that the use of gonadotrophins increased both SSR rate (*P* < 0.05) and production of sperm into the ejaculate (*P* < 0.05) compared to no hormone therapy. Similarly, Hussein *et al.* (2013) studied multiple hormone therapy agents (SERMs, gonadotrophins and a combination of SERMs and gonadotrophins) and reported that hormone therapy increased both SSR rate (*P* < 0.05) and production of sperm into the ejaculate (*P* < 0.05) compared to the control group not receiving any treatment. The cause for the differences in outcomes reported in the literature is unclear but may be related to differences in patient cohorts and treatment regimens and durations.

The following adverse effects were reported with the use of hormone therapy: loss of libido, cutaneous rash, anxiety and a paradoxical decline in testosterone levels.

The main limitation to the current evidence is the lack of standardization in terms of patient cohorts, treatment regimens and outcome reporting, with few studies report pregnancy or live birth rates and a large proportion of the data being retrospective, case series.

### Studies including men with eugonadal and hypergonadotropic hypogonadal non-obstructive azoospermia

Two case series (Kumar *et al.*, 1990; Kobori *et al.*, 2015) have investigated the use of hormone therapy in a mixed cohort of NOA men with hypergonadotropic hypogonadism and eugonadism. One study solely utilized gonadotrophin therapy (Kobori *et al.*, 2015) and one study investigated the use of either gonadotrophin or clomiphene citrate use (Kumar *et al.*, 1990). Both studies reported the rate of sperm production in the ejaculate and excluded chromosomal abnormalities. No adverse effects were reported in either of the studies. The effects of hormone therapy on the production of sperm in the ejaculate were inconsistent between studies and both studies were limited because the data was retrospective and lacked control cohorts.

### Meta-analysis

For the meta-analysis, we only included controlled studies and, owing to the limited number of studies, we pooled data for all hormone classes. Hence, no analysis was performed on the individual drug classes.

Of the retrieved texts, we analysed 10 studies (Tables I and II). Among them, five studies (Reifsnyder *et al.*, 2012; Shiraishi *et al.*, 2012; Majzoub *et al.*, 2016; Hu *et al.*, 2018; Amer *et al.*, 2020) included hypergonadotropic subjects whereas five (Aydos *et al.*, 2003; Hussein *et al.*, 2013; Gul, 2016; Cocci *et al.*, 2018; Sen *et al.*, 2020) included normogonadotropic men. The characteristics of the retrieved studies are reported in Tables I and II. The retrieved studies included 985 patients with a mean (±SD) age of 31.9 ± 4.2 years and a mean follow-up of 17.2 ± 9.4 weeks. The modality of treatment and the drug dosages differed among studies (Tables I and II).

The *I*^2^ in trials assessing overall SSR was 58.2 (*P* < 0.01). A funnel plot and Begg adjusted rank correlation test (Kendall’s *τ*: 0.00 *P* = 1.00) was non-significant suggesting publication bias was not present. Figure 2 demonstrates the standard error of sperm retrieval rate by Mantel–Haenszel log odds ratio.

**Figure 3.**
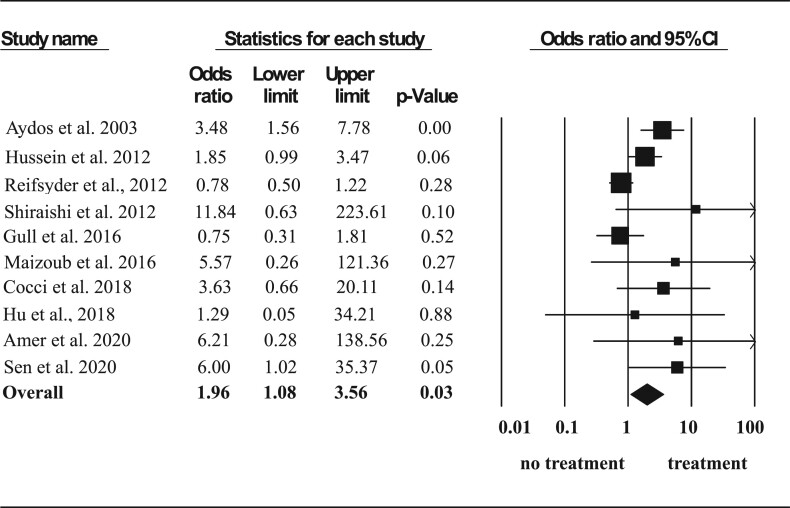
**Effect of hormone therapy on surgical sperm retrieval rate in men with non-obstructive azoospermia.** A Forest plot demonstrating the individual and cumulative odds ratios for surgical sperm retrieval.

Overall, a higher SSR in subjects pre-treated with hormone therapy was observed (odds ratio (OR) 1.96, 95% CI: 1.08–3.56, *P* = 0.03) (Fig. 3).

**Figure 4.**
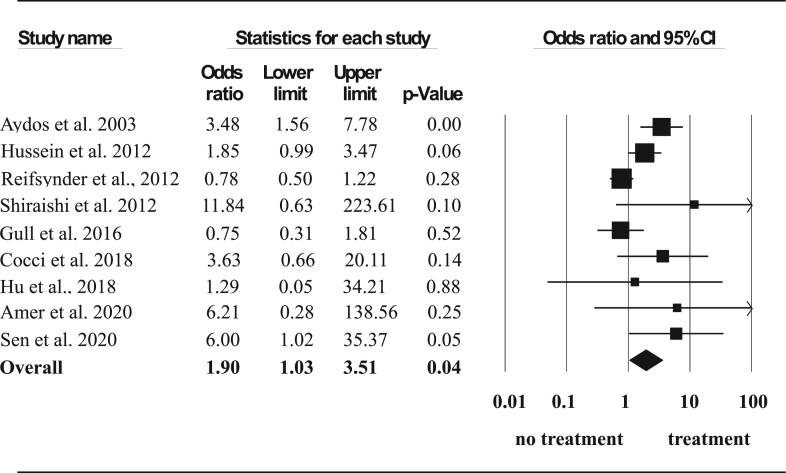
**Effect of hormone therapy on surgical sperm retrieval rate, including only patients with Klinefelter syndrome.** A Forest plot demonstrating the individual and cumulative odds ratios for surgical sperm retrieval. This analysis excluded the study by Majzoub *et al.* (2016). We excluded this study, as it only included Klienfelter syndrome patients and we wanted to see if this disproportionately affected the results and thus whether are results would be applicable to a non-Klienfelter population.

Sensitivity analysis, excluding one study enrolling only patients with Klinefelter syndrome (Majzoub *et al.*, 2016), confirmed the previous observation that hormone therapy was associated with a higher SSR (OR 1.90, 95% CI: 1.03–3.51, *P* = 0.04) (Fig. 4).

**Figure 5.**
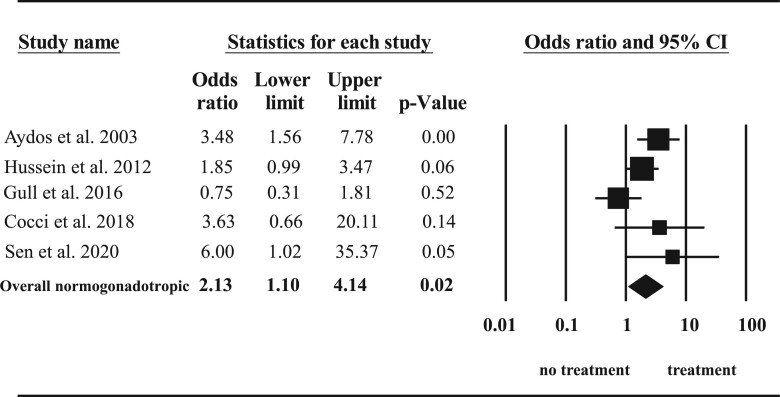
**Effect of hormone therapy on surgical sperm retrieval rate in normogonadotropic men with non-obstructive azoospermia.** A Forest plot demonstrating the individual and cumulative odds ratios for surgical sperm retrieval.

**Figure 6.**
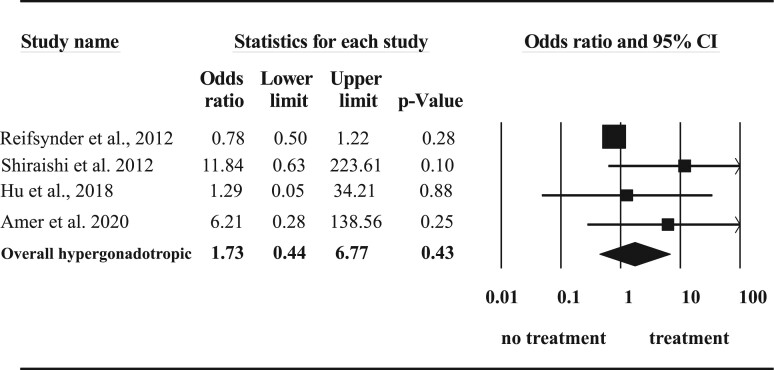
**Effect of hormone therapy on surgical sperm retrieval rate in hypergonadotropic men with non-obstructive azoospermia.** A Forest plot demonstrating the individual and cumulative odds ratios for surgical sperm retrieval.

Further subgroup analysis of baseline hormone status demonstrated only a significant improvement in normogonadotropic men (OR 2.13, 95% CI: 1.10–4.14, *P* = 0.02) (Fig. 5) but not in hypergonadotropic subjects (OR 1.73, 95% CI: 0.44–6.77, *P* = 0.43) (Fig. 6).

Finally, when the only study not published as a full text (Sen *et al.*, 2020) was excluded, there was a non-statistically significant trend towards a higher SSR in the normogonadotropic group compared to the hypergonadotropic cohort (OR 1.9, 95% CI: 0.95–3.78, *P* = 0.07).

### Risk of bias

The risk of bias analysis is demonstrated in Tables IV and V. A limitation to the data was that none of the studies were randomized and most of the evidence was at risk of confounding bias. The main merits of the literature were that there was a low risk of bias from missing data. Overall, six of the studies were categorized as being of serious risk of bias and four studies of moderate risk of bias.

**Table IV:** Risk of bias for studies included in the meta-analysis that investigated eugonadal men with non-obstructive azoospermia.

Risk of bias									
Study name (year)	Study design	Confounding	Patient selection	Interventions classification	Deviation form intended interventions	Missing data	Measurement outcomes	Selection of reported result	Outcome
Aydos *et al.* (2003)	Case control	Serious	Low	Low	Low	Low	Moderate	Moderate	Serious
Cocci *et al.* (2018)	Case control	Serious	Low	Moderate	Low	Low	Serious	Low	Serious
Gul (2016)	Case control	Moderate	Moderate	Serious	Low	Low	Serious	Low	Serious
Hussein *et al* (2013)	Case control	Serious	Serious	Serious	Moderate	Low	Serious	Moderate	Serious

**Table V:** Risk of bias for studies included in the meta-analysis that investigated men with non-obstructive azoospermia and hypergonadotropic hypogonadism.

Risk of bias									
Study name (year)	Study design	Confounding	Patient selection	Interventions classification	Deviation form intended interventions	Missing data	Measurement outcomes	Selection of reported result	Outcome
Hu *et al.* (2018)	Case control	Low	Low	Low	Low	Low	Moderate	Moderate	Moderate
Shiraishi *et al* (2012)	Case control	Moderate	Low	Low	Moderate	Low	Low	Low	Moderate
Reifsnyder *et al.* (2012)	Case control	Serious	Serious	Moderate	Moderate	Low	Low	Moderate	Serious
Majzoub *et al* (2016)	Case control	Moderate	Low	Low	Low	Low	Moderate	Moderate	Moderate
Sen *et al.* (2020)	Case control	Serious	Low	Low	Low	Low	Moderate	Low	Serious
Amer *et al* (2020)	Case control	Moderate	Low	Low	Low	Low	Moderate	Low	Moderate

## Discussion

This is the first systematic review and meta-analysis investigating hormone stimulation therapy in men with NOA and either primary hypogonadism or normal hormone status.

Currently, there are no established pharmacological therapies to treat NOA in men with primary hypogonadism, while rates of successful SSR have been reported to be only 47% (Corona *et al.*, 2019). Within this context, hormone therapies have been used empirically by reproductive clinicians to improve the chances of sperm retrieval, although there are limited large-scale RCTs supporting this in clinical practice. There is a theoretical rationale (Tharakan *et al.*, 2020) to the use of hormone therapy prior to a TESE, as ITT is required for spermiogenesis and human studies have observed that hormone therapy can increase ITT (Shinjo *et al.*, 2013). A study comparing men with hypergonadotropic hypogonadism NOA to those with obstructive azoospermia observed that the former group had more testicular interstitial fibrosis than the latter and the use of hCG was associated with a reduction in fibrotic areas (Oka *et al.*, 2017). However, it remains unclear as to the optimal level of ITT to facilitate spermatogenesis and improve SSR. Moreover, the measurement of ITT requires testicular aspiration, which is an invasive procedure and there is a poor correlation between serum testosterone and ITT levels (Tharakan *et al.*, 2020). A transgenic murine study suggested that an increase of FSH may also contribute to stimulation of spermatogenesis despite a low ITT (Oduwole *et al.*, 2018b); however, this needs to be validated by further data, and the optimal level of FSH elucidated, especially given that another transgenic mice study (Allan *et al.*, 2004) reported that FSH stimulation alone was unable to produce complete spermatogenesis. Therefore, many clinicians have utilized hormone therapy empirically given the theoretical plausibility and lack of alternative treatments. However, the available literature is of low-quality evidence with an abundance of retrospective case series, with only one RCT and a small number of case-controlled studies. Furthermore, we observed moderate heterogeneity (*I*^2^ = 58.2, *P* < 0.01) in the meta-analysis data. The current literature is inconsistent in terms of therapies, duration of treatment, patient cohorts (genetic status, mixed cohorts of oligospermic men and men with NOA) and surgical techniques (primary versus secondary TESE). Moreover, several studies had missing data, with particular reference to post-treatment hormone levels and adverse events outcomes. Furthermore, because a wide range of treatment regimens were utilized, the optimal hormone therapy or duration of treatment to optimize SSR rates remains unclear.

Within these limitations, our meta-analysis demonstrated that, overall, hormone therapy significantly improved SSR (OR 1.96, *P* = 0.03). Given the paucity of controlled studies, we were unable to perform a sub-analysis on the individual hormone therapy classes. However, when stratifying by baseline hormone status, the effect of hormone therapy on SSR was only seen in men with normal gonadotrophin levels and not in those who were hypergonadotropic. The underlying mechanisms for this are unclear but could be related to the fact that hypergonadotropic hypogonadism may reflect a more severe form of disease with irreversible damage to spermatogenesis and hence is a condition refractory to hormone therapy. Furthermore, in this subset of patients FSH levels are already increased and therefore further hyperstimulation is likely to have less pronounced effects on spermatogenesis. However, there are currently no animal or human studies in the literature to validate this theory.

Murine studies have demonstrated differential endocrinological and reproductive outcomes from the disruption of the androgen receptor in different cell types of the testes. Wang *et al.* (2009) reported that cell-specific androgen receptor knockout in germ cells resulted in normal gonadotrophin and testosterone levels, testicular size, sperm count and fertility. However, cell-specific androgen receptor knockout in Leydig cells was associated with hypergonadotropic hypogonadism, decreased testicular size and azoospermia. Extrapolating this data to our study, these findings suggest that androgen receptor polymorphisms could also be responsible for the different endocrinological and reproductive characteristics of NOA and may also affect the response to hormone therapy. Moreover, there is data showing that polymorphisms in the FSHR may affect hormone profiles (Lindgren *et al.*, 2012), sperm parameters (Lindgren *et al.*, 2012) and contribute to different responses to hormone therapy (Selice *et al.*, 2011). Lindgren *et al.* (2016) reported that men homozygous for the Thr307Thr/Asn680Asn single-nucleotide polymorphism combination had a significantly lower FSH (*P* = 0.009) and total testosterone level (*P* < 0.0001) but a higher sperm concentration (*P* = 0.040) and testicular volume (*P* = 0.002) compared with carriers of other FSHR variants. Selice *et al.* (2011) observed that the use of FSH therapy only conferred to a statistically significant improvement of sperm parameters in oligospermic men who were homozygote Ala307-Ser680/Ala307-Ser680 or had heterozygote Thr307-Asn680/Ala307-Ser680 common allelic variants. These studies suggest that the effects of hormone therapy may be dependent on genetic alterations in the androgen receptor or FSHR but further studies specifically investigating non-azoospermic men and the effects on SSR rates are needed.

We observed that all identified controlled studies had moderate to serious risk of bias (Tables IV and V). Therefore, although our findings have suggested that hormone therapy may be beneficial in eugonadal NOA men, it is based on low-quality evidence with a significant risk of bias. The current literature is also deficient with regards to information pertaining to the costs of different hormone treatments. Furthermore, no study reported on the prevalence of hypogonadal symptoms in their study cohorts. This would be a useful parameter to assess, as it could potentially justify the use of hormonal manipulation for the dual benefits of infertility and symptomatic male hypogonadism. Moreover, few studies have included data on pregnancy and live birth rates, which is needed to understand how hormone therapy may ultimately influence the quality of sperm and ART outcomes. Therefore, we would not recommend hormone stimulation therapy outside of clinical trials.

There were several limitations to this study. Most of the studies were not randomized or prospective and do not report study participation rate. Thus, the findings of the meta-analysis should be treated with caution given the high risk of selection bias. Furthermore, different hormone assays were utilized presenting a further source of bias. In addition to this, SSR outcomes are influenced by both surgical and embryological factors, including the type of surgery (Bernie *et al.*, 2015), experience of the surgeon (Ishikawa *et al.*, 2010), and the methods used to process the sperm from testicular tissue (Crabbé *et al.*, 1998). Furthermore, many of these studies are not consistent in standardized reported outcomes such as surgical technique used and quantity and quality of sperm retrieved. Available data did not allow us to correct for any of these confounding factors. Moreover, another prognostic factor to sperm retrieval surgery is histopathological subtype (Flannigan *et al.*, 2017), although most studies did not report data pertaining to this confounding variable. However, it must be noted that it is common for NOA patients to have a mixed histopathological pattern (McLachlan *et al.*, 2007). We were unable to provide any analysis regarding aetiology and its effects on SSR, which represents a further limitation (e.g. some genetic or acquired conditions, such as azoospermia factor microdeletions, confer a worse prognosis for SSR outcomes (Kamp *et al.*, 2001)). In most studies, there were no comparison of markers of testicular function (such as testicular size, and Leydig and Sertoli cellular secretory function parameters: insulin like three peptide, inhibin B and anti-Müllerian hormone) and therefore this study was unable to exclude these confounding factors.

## Conclusion

This systematic review and meta-analysis observed that the current literature pertaining to hormone stimulation in men with NOA provides low-quality evidence and is at moderate or severe risk of bias. Within these limitations, hormone therapy overall appears to increase SSR rate but only in men with NOA and normal gonadotrophin status. However, there is a paucity of controlled trials to provide any evidence-based recommendations, and no firm inferences can be provided given the poor quality of the data. Moreover, many studies do not report adverse events. Therefore, based upon the current literature we cannot advocate the use of hormone therapy in men with NOA until further high powered, RCTs are performed.

## Data Availability

The data underlying this article will be shared on reasonable request to the corresponding author.
